# Impact of dexmedetomidine supplemented analgesia on delirium in patients recovering from orthopedic surgery: A randomized controlled trial

**DOI:** 10.1186/s12871-021-01441-3

**Published:** 2021-09-13

**Authors:** Hong Hong, Da-Zhi Zhang, Mo Li, Geng Wang, Sai-Nan Zhu, Yue Zhang, Dong-Xin Wang, Daniel I. Sessler

**Affiliations:** 1 Departments of Anesthesiology and Critical Care Medicine, Peking University First Hospital, Beijing, 100034 China; 2 Department of Anesthesiology, Beijing Jishuitan Hospital, Beijing, China; 3 Department of Biostatistics, Peking University First Hospital, Beijing, China; 4 Peking University Clinical Research Institute, Shenzhen, China; 5 Outcomes Research Consortium, Cleveland, OH USA; 6 Department of Outcomes Research, Cleveland Clinic, Cleveland, OH USA

**Keywords:** Elderly, Orthopedic Procedures, Delirium, Dexmedetomidine, Postoperative Period

## Abstract

**Background:**

Dexmedetomidine promotes normal sleep architecture; the drug also improves analgesia. We therefore tested the hypothesis that supplementing intravenous analgesia with dexmedetomidine reduces delirium in older patients recovering from orthopedic surgery.

**Methods:**

In this double-blinded randomized controlled trial, we enrolled 712 older (aged 65–90 years) patients scheduled for major orthopedic surgery. Postoperative analgesia was provided by patient-controlled intravenous sufentanil, supplemented by randomly assigned dexmedetomidine (1.25 μg/mL) or placebo, for up to three days. The primary outcome was the incidence of delirium assessed twice daily with the Confusion Assessment Method. Among secondary outcomes, pain severity was assessed twice daily and sleep quality once daily, each with an 11-point scale where 0 = no pain/the best possible sleep and 10 = the worst pain/the worst possible sleep.

**Results:**

The incidence of postoperative delirium was 7.3% (26 of 354) with placebo and 4.8% (17 of 356) with dexmedetomidine; relative risk 0.65, 95% CI 0.36 to 1.18; *P* = 0.151. Dexmedetomidine reduced pain both at rest (median difference -1 to 0 points, *P* ≤ 0.001) and with movement (-1 points, *P* < 0.001) throughout the first 5 postoperative days; it also improved subjective sleep quality during the first 3 postoperative days: day one median difference -1 point (95% CI -1 to 0), *P* = 0.007; day two 0 point (-1 to 0), *P* = 0.010; and day three 0 point (-1 to 0), *P* = 0.003. The incidence of adverse events was similar in each group.

**Conclusions:**

Supplementing sufentanil intravenous analgesia with low-dose dexmedetomidine did not significantly reduce delirium, but improved analgesia and sleep quality without provoking adverse events.

**Trial registration:**

www.chictr.org.cn: ChiCTR1800017182 (Date of registration: July 17, 2018); ClinicalTrials.gov:NCT03629262 (Date of registration: August 14, 2018).

**Supplementary Information:**

The online version contains supplementary material available at 10.1186/s12871-021-01441-3.

## Background

Because the population is aging, the demand for orthopedic surgery is increasing, especially among the elderly. Delirium is common amongst elderly patients recovering from major orthopedic surgery. The reported incidence ranges from 5 to 14% after total joint arthroplasty [1], from 0.5 to 24% after spinal surgery [2], and from 12 to 56% after hip fracture surgery [3]. Patients who experience postoperative delirium have worse outcomes including prolonged hospitalization, increased costs, lower odds of home discharge, more readmissions, delayed functional recovery, and increased perioperative and long-term mortality [4, 5]. While delirium is now recognized as a serious complication, there is so far no convincing evidence that any preventive strategy is effective [6].

Postoperative delirium is probably facilitated by multiple factors which may include severe pain [7], opioid medication [8], sleep disturbances [9], and the stress response and inflammation consequent to surgical tissue injury [10]. Dexmedetomidine is a highly selective alpha-2-adrenergic agonist with sedative, analgesic, and anxiolytic properties. For postoperative patients, low-dose dexmedetomidine infusion promoted normal sleep architecture by increasing total and stage 2 non-rapid eye movement sleep but not rapid eye movement sleep [11]. When used in combination with opioids after surgery, it improves analgesia and sleep quality while reducing opioid consumption [12, 13]. Dexmedetomidine also attenuates the surgical stress response and consequent inflammation [14]. These characteristics make dexmedetomidine a potential candidate for delirium prevention. Indeed, when used during anesthesia or in the intensive care unit, dexmedetomidine reportedly reduces postoperative delirium [15].

We therefore tested the primary hypothesis that supplementing intravenous analgesia with dexmedetomidine reduces delirium in elderly patients recovering from major orthopedic surgery. Secondarily, we tested the hypotheses that dexmedetomidine supplementation improves postoperative analgesia and subjective sleep quality.

## Methods

This randomized, double-blinded, placebo-controlled trial with two parallel groups was performed at the Peking University First Hospital and Beijing Jishuitan Hospital, both in Beijing, China. The study protocol was approved by the Biomedical Research Ethics Committee of Peking University First Hospital (2018–131 on July 18, 2018; No.6 Da-Hong-Luo-Chang Street, Beijing 100,034, China; Chairperson Prof. Xiao-Hui Guo) and the Ethics Committee of Beijing Jishuitan Hospital (201,808–06 on August 28, 2018; No.31 Xin-Jie-Kou East Street, Beijing 100,035, China; Chairperson Prof. Xiao-Lan Zhao). Written informed consents were obtained from all patients or their legal representatives. The trial was registered prior to patient enrolment at www.chictr.org.cn (ChiCTR1800017182; principal investigator: Dong-Xin Wang; date of registration: July 17, 2018) and ClinicalTrials.gov (NCT03629262; principal investigator: Dong-Xin Wang; date of registration: August 14, 2018). This manuscript adheres to the applicable Consolidated Standards of Reporting Trials (CONSORT) guidelines.

Potential participants were screened and consented pre-operatively. We included patients aged 65–90 years who were scheduled for elective hip or knee arthroplasties, hip fracture repair, or spinal surgery and who agreed to use patient-controlled intravenous analgesia postoperatively. We excluded patients who: (1) were scheduled for cancer surgery; (2) had a pre-operative history of schizophrenia, epilepsy, parkinsonism, or myasthenia gravis; (3) inability to communicate due to coma, profound dementia, or language barrier; (4) sick sinus syndrome, severe sinus bradycardia (< 50 beats per min), or second- or third-degree atrioventricular block without a pacemaker; (5) diagnosed sleep apnea syndrome or a STOP-Bang score ≥ 3 combined with a serum bicarbonate ≥ 28 mmol.l^−1^; or, (6) severe hepatic dysfunction (Child–Pugh class C), renal failure (requiring dialysis before surgery), American Society of Anesthesiologists physical status > IV, or estimated survival ≤ 24 h.

### Protocol

Hip and knee arthroplasties were performed with neuraxial anesthesia or a peripheral nerve block, either combined with general anesthesia. Neuraxial anesthesia included epidural and combined spinal-epidural anesthesia. Peripheral nerve blocks included lumbar plexus, sciatic nerve, femoral nerve, and iliac fascial space. All were performed with ultrasound guidance. Patients were sedated during block insertion with dexmedetomidine and/or midazolam. The target was to maintain Richmond Agitation-Sedation Scale (RASS) scores between -2 and 0. RASS scores range from –5 (unarousable) to + 4 (combative), with 0 indicating an alert and calm subject [16].

Spinal anesthesia was performed with bupivacaine; epidural anesthesia and peripheral nerve blocks used ropivacaine. Per routine, epidural catheters, if used, were withdrawn at end of surgery because patients were given prophylactic antithrombotic therapy after surgery. Regional analgesia was not used in spine surgery patients.

About a third of participating patients had general anesthesia alone, and a small fraction had general anesthesia combined with epidural anesthesia or peripheral nerve block. General anesthesia was induced with midazolam (1–3 mg), propofol or etomidate and sufentanil or remifentanil, and maintained with propofol infusion, sevoflurane and/or nitrous oxide inhalation, and sufentanil or remifentanil. Anesthetic drugs were adjusted to maintain Bispectral Index between 40 and 60. The Bispectral Index is an electroencephalographic measure of hypnotic depth, ranging from 0 to 100, with values between 40 and 60 considered optimal.

Random numbers were computer-generated in a 1:1 ratio with a block size of 4 using SAS 9.2 software (SAS Institute, Cary, NC, USA). Randomization was stratified by trial site and surgical location (hip or knee versus spine). Trial drugs, either dexmedetomidine 200 μg/2 ml or a comparable volume of 0.9% saline, were provided as clear aqueous solutions in identical appearing 3-ml ampules (Yangtze River Pharmaceutical Group Co., Ltd., Jiangsu, China). Sequential randomization numbers were assigned to vials by a pharmacist who was otherwise not involved in the trial. Allocation was concealed in sequentially numbered sealed opaque envelopes until the end of the trial. All investigators, clinicians, and patients were therefore completely blinded to treatment allocation. But in case of emergency (such as unexpected, rapid deterioration in a participant’s clinical status), clinicians could adjust or stop drug administration if deemed clinically necessary. Unmasking was allowed only if clearly needed for clinical purposes.

Postoperative analgesia was primarily provided by patient-controlled intravenous administration of the trial drug (either dexmedetomidine 200 μg or 0.9% saline) and 200 μg sufentanil, diluted with 0.9% saline to 160 ml. The patient-controlled pump was programmed to deliver 2-ml boluses with a lockout interval of 8 min and a background infusion of 1 ml.h^−1^. We adopt this dosing regimen because it has been safely used in our clinical practice and our previous studies [13]. Patient-controlled analgesia was continued for at least 24 h, but not longer than 72 h after surgery. Other analgesics including non-steroidal anti-inflammatory drugs, acetaminophen, and opioids were administered when the Numeric Rating Scale (NRS, an 11-point scale where 0 indicates no pain and 10 the worst pain) of pain remained > 3 despite self-controlled analgesia. As a routine practice, patient-controlled analgesia was stopped after 48 h by anesthesia or ward nurses when the NRS pain score with movement was ≤ 3, analgesics could be taken orally, and/or hospital discharge was planned. Open-label dexmedetomidine was not allowed except for treatment of delirium.

Patients were transferred to the intensive care unit (ICU) when clinically indicated; otherwise, they remained in the post-anesthesia care unit for at least 30 min, and were then sent to a surgical ward. Electrocardiogram, invasive or non-invasive blood pressure, and pulse oxygen saturation were monitored continually in critical care and recovery units. Non-invasive blood pressure and pulse oxygen saturation were monitored intermittently until next morning. Non-invasive blood pressure and heart rate were then monitored once or twice daily until hospital discharge. Those with unstable hemodynamic were monitored frequently and transferred to an intensive care unit if necessary.

Non-pharmacological strategies to reduce delirium, including restoring hearing and vision aids, reorientation, cognitive stimulation, early mobilization, sleep-promotion and timely correction of dehydration were all used per clinical routine [17]. Patients with delirium were initially managed with non-pharmacological measures and treatment of primary diseases. Severe agitation (RASS score of + 3 or more) was treated with haloperidol and/or dexmedetomidine [18].

### Measurements

Baseline data included demographic characteristics, surgical diagnosis, pre-operative comorbidities, surgical history, smoking and alcohol consumption, and pre-operative medications and laboratory test results. The Charlson comorbidity index was calculated [19]. During the pre-operative interview, cognitive function was evaluated with the Mini-Mental State Examination score (MMSE; scores range from 0 to 30, with higher scores indicating better cognitive function) [20].

Routine intra-operative monitoring included electrocardiogram, non-invasive blood pressure, pulse oxygen saturation and urine output. We also recorded Bispectral Index, end-tidal carbon dioxide partial pressure, and volatile anesthetic concentration. Intra-arterial and central venous pressures were monitored when clinically indicated. Other intra-operative data included the type and duration of anesthesia, types and doses of medication during anesthesia, type and duration of surgery, estimated blood loss, administered fluid volumes, and blood transfusions. Postoperative data included intensive care unit admission after surgery, study drug and sufentanil consumption during patient-controlled analgesia, supplemental analgesics and hypnotics within 5 days, and other medications.

Postoperative pain severity was assessed twice daily, between 8–10 AM and between 6–8 PM, with the NRS, both at rest and with movement. “Movement” was defined as turning over on/getting off the bed for patients after spinal surgery and flexion–extension/rehabilitation exercise for those after joint surgery. The most severe pain score during movement was recorded. Subjective sleep quality was assessed once daily, between 8–10 AM, with the NRS. Patients were asked to give a comprehensive score that best evaluate their overall sleep quality last night, i.e., a good night’s sleep or a bad night’s sleep. The scale ranged from 0 to 10, with 0 representing the best possible sleep and 10 the worst possible sleep. A minimum difference of 1 point was considered clinically meaningful [21].

Our primary outcome was delirium which was assessed twice daily, between 8–10 AM and between 6–8 PM, with the Confusion Assessment Method (CAM) in patients who were not intubated or the CAM for the Intensive Care Unit (CAM-ICU) in patients who were intubated [22, 23]. Delirium assessments continued until the 5^th^ postoperative day or hospital discharge, whichever occurred first. Immediately before assessing delirium, patients’ sedation or agitation status was assessed with the RASS. When patients were deeply sedated or unarousable (RASS score –4 or –5), they were considered to be comatose and delirium was not assessed. In patients with positive CAM assessments, delirium was classified into three motoric subtypes: (1) hyperactive (RASS score was consistently positive, + 1 to + 4); (2) hypoactive (RASS score was consistently neutral or negative, –3 to 0); and, (3) mixed [24].

Secondary outcomes included pain, subjective sleep quality, and RASS score during the first 5 days; postoperative opioid consumption within 5 days; postoperative duration of hospitalization; postoperative complications within 30 days; 30-day mortality; and cognitive function and quality-of-life in 30-day survivors. Sufentanil equivalent dose was calculated in order to compare opioid consumption [25]. Postoperative complications were defined as newly occurred adverse conditions that required therapeutic intervention; that is, class 2 or higher by Clavien-Dindo classification.

Thirty days after surgery, cognitive function was assessed with the Chinese version Telephone Interview for Cognitive Status-modified (TICS-m; scores range from 0 to 48, with higher scores indicating better function) [26]. Quality-of-life was assessed with the World Health Organization Quality of Life-brief version, WHOQOL-BREF; a 24-item questionnaire that provides assessments of the quality of life in physical, psychological, and social relationship, and environmental domains. For each domain, the score ranges from 0 to 100, with higher score indicating better function; minimal important difference 0.5 SD [27].

Adverse events were monitored from the beginning of patient-controlled analgesia until 72 h after surgery. Among anticipated abnormalities, we defined bradycardia as heart rate < 45 beats per minute, hypotension as systolic blood pressure < 90 mmHg or a decreased of more than 30% from baseline, tachycardia as heart rate > 100 beats per minute, hypertension as systolic blood pressure > 180 mmHg or an increase of more than 30% from baseline, and hypoxemia as pulse oxygen saturation < 90%.

Pre-operative interview and postoperative follow-up were performed by two qualified investigators (HH and ML) who did not participate in anesthesia or perioperative care. Both investigators were trained to follow the study protocol, and in use of the CAM and the CAM-ICU by a psychiatrist. During the training process, the symptoms, diagnosis and treatment of delirium were presented, the uses of the CAM and the CAM-ICU were explained, and simulation training courses on patient-actors were performed and continued until the diagnosis of delirium reached 100% agreement between the investigators and the psychiatrist. The training process was repeated every 4–6 months throughout the trial.

### Sample size estimation

Based on previous results [28, 29], we expected that delirium would occur in 12.5% of elderly patients after orthopedic surgery in the placebo group. In a recent trial, low-dose dexmedetomidine reduced postoperative delirium by about 60% [30]. We assumed that delirium would be reduced by 50% in the dexmedetomidine group. With significance set at 0.05 and power set at 80%, the sample size was 676 patients. Anticipating about 5% loss-to-follow-up, we planned to enroll 712 patients. Sample size was calculated with the PASS 11.0 software (Stata Corp. LP, College Station, TX, USA).

### Statistical analysis

The balance of baseline data between groups was assessed using absolute standardized difference, calculated as the absolute difference in means, medians, or proportions divided by the pooled standard deviation [31]. Baseline variables with an absolute standardized difference ≥ 0.147 (i.e.,$1.96 \times \sqrt {{{\left( {{\text{n}}1 + {\text{n}}2} \right)} \mathord{\left/ {\vphantom {{\left( {{\text{n}}1 + {\text{n}}2} \right)} {\left( {{\text{n}}1 \times {\text{n}}2} \right)}}} \right. \kern-\nulldelimiterspace} {\left( {{\text{n}}1 \times {\text{n}}2} \right)}}}$) were considered imbalanced and would be adjusted for in all analyses when considered necessary.

The primary outcome, i.e., the incidence of delirium within 5 days after surgery, was compared with chi square tests, with differences between groups expressed as relative risk (95% CI). For patients who were discharged or died within 5 days, the last delirium assessment results were used to replace the missing data when calculating incidence within 5 days; missing data were not replaced when calculating daily prevalence of delirium. The interactions between treatment effect and predefined factors were assessed separately with logistic regression models.

Other numeric variables were analyzed using independent-sample *t* or Mann–Whitney U tests. Differences (and 95% CIs for the differences) between medians were calculated with Hodges-Lehmann estimators. Categorical variables were analyzed using chi square, continuity-corrected chi square, or Fisher exact tests. Ordinal data were assessed by Mann Whitney U tests. Time-to-event variables were evaluated with Kaplan–Meier estimators, with differences between groups assessed with log-rank tests. Patients who died within 30 days were censored at the time of death; and those who stayed in hospital for longer than 30 days were censored at 30 days after surgery. Missing data were not replaced.

Outcome analyses were performed in the intention-to-treat population. For the primary outcome, a per-protocol analysis was also performed. Differences were calculated as dexmedetomidine group *vs.* or minus placebo group. No interim analysis was planned. The trial stopped when planned sample size was reached. For all hypotheses, two-tailed *P* values < 0.05 were considered statistically significant. For the interactions between treatment effect and predefined factors, *P* values < 0.10 were considered statistically significant. Statistical analyses were performed on SPSS 25.0 software package (IBM SPSS, Chicago, IL).

## Results

**Fig. 1:**
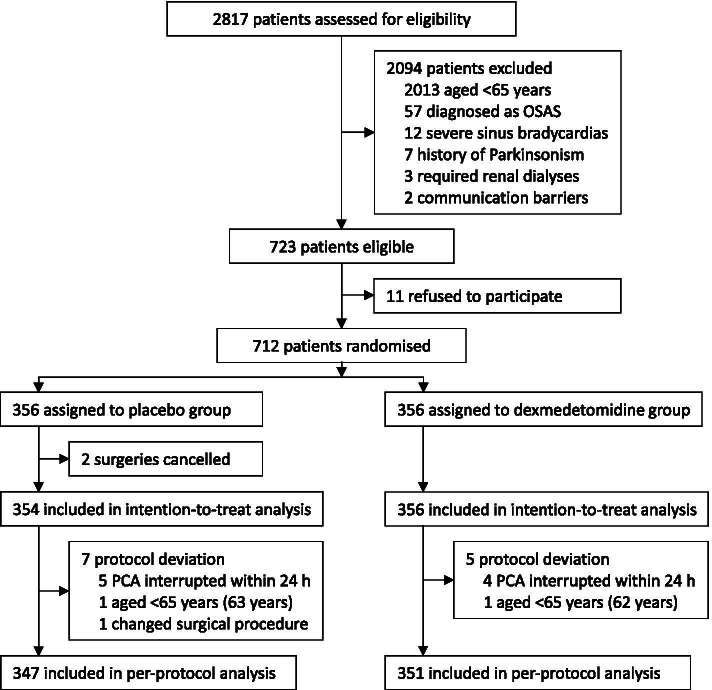
Trial diagram. OSAS, obstructive sleep apnea syndrome; PCA, patient-controlled analgesia

The trial started on October 28, 2018. From that time to December 6, 2019, 2,817 patients were screened for eligibility, among whom 712 patients were enrolled and randomly assigned to receive either dexmedetomidine (*n* = 356) or placebo (*n* = 356). Surgeries were cancelled in 2 patients, protocol deviation occurred in 12 patients. Specifically, study drug administration was modified in 9 patients, age was < 65 years in 2 patients, the surgical procedure changed in 1 patient. There was never need to unmask the study drug, and no assessment was aborted due to deep sedation. A total of 710 patients who were randomized and underwent surgeries were included in the intention-to-treat analysis. Per-protocol analysis included 698 patients who completed the study according to the established protocol (Fig. 1). Thirty-day follow-up of the last participant finished on January 5, 2020.

The two groups were well balanced on baseline characteristics (Table 1). Intra-operative variables were similar in each group, as were postoperative sufentanil use and use of supplemental analgesics, hypnotics, and antiemetics over the initial 5 postoperative days. In patients assigned to dexmedetomidine, the mean infusion rate was 0.026 μg·kg^−1^·h^−1^ (Table 2).

**Table 1:** Baseline data

	Placebo group (*n* = 354)	Dexmedetomidine group (*n* = 356)	ASD
Age (year)	71 ± 5	71 ± 5	0.042
Male sex	113 (31.9%)	116 (32.6%)	0.014
Body mass index (kg/m)	26.1 ± 3.5	26.0 ± 3.6	0.042
Education (year)	8.2 ± 4.5	8.3 ± 4.9	0.018
Preoperative comorbidity			
Stroke	36 (10.2%)	37 (10.4%)	0.007
Hypertension	188 (53.1%)	184 (51.7%)	0.028
Coronary heart disease	35 (9.9%)	29 (8.1%)	0.064
Arrhythmia	15 (4.2%)	9 (2.5%)	0.109
COPD	1 (0.3%)	1 (0.3%)	** < **0.001
Diabetes mellitus	86 (24.3%)	71 (19.9%)	0.109
History of surgery	208 (58.8%)	183 (51.4%)	0.141
Chronic smoking ^a^	32 (9.0%)	37 (10.4%)	0.044
Alcoholism ^b^	23 (6.5%)	27 (7.6%)	0.041
Preoperative medication			
Calcium channel blockers	99 (28.0%)	95 (26.7%)	0.029
ACEI/ARB	64 (18.1%)	73 (20.5%)	0.060
Beta‐blockers	26 (7.3%)	24 (6.7%)	0.024
Insulin for diabetes mellitus	24 (6.8%)	18 (5.1%)	0.079
Chronic use of benzodiazepine	8 (2.3%)	11 (3.1%)	0.048
Preoperative laboratory tests			
Hematocrit (%)	40 ± 4	40 ± 4	0.064
Albumin (g/L)	42 ± 4	42 ± 4	0.048
Creatinine (μmol/L)	69.5 ± 16.8	69.3 ± 18.0	0.023
Glucose < 4.0 or > 10.0 mmol/ L	18 (5.1%)	10 (2.8%)	0.138
Sodium < 135.0 or > 145.0 mmol/L	25 (7.1%)	27 (7.6%)	0.020
Potassium < 3.5 or > 5.5 mmol/L	13 (3.7%)	19 (5.3%)	0.074
Charlson Comorbidity Index (score)	0 (0 to 1)	0 (0 to1)	0.041
ASA classification			0.031
I	8 (2.3%)	3 (0.8%)	
II	278 (78.5%)	293 (82.3%)	
III	67 (18.9%)	60 (16.8%)	
IV	1 (0.3%)	0 (0.0%)	
Preoperative evaluation			
MMSE (score) ^c^	26.1 ± 3.0	26.1 ± 2.9	0.003
NRS of pain, at rest (score) ^d^	1 (0 to 2)	0 (0 to 2)	0.009
NRS of pain, with movement (score) ^d^	5 (3 to 6)	4 (3 to 6)	0.105
NRS of sleep quality (score) ^e^	2 (1 to 3)	2 (1 to 3)	0.083
Hypnotics in preoperative night	24 (6.7%)	20 (5.6%)	0.043
Study site			0.005
Centre 1	210 (59.3%)	212 (59.6%)	
Centre 2	144 (40.7%)	144 (40.4%)	

Data are mean ± SD, number (%) or median (interquartile range)

*ASD* absolute standardized difference (an ASD of ≥ 0.147 is considered imbalanced between the two groups), *COPD* chronic obstructive pulmonary disease, *ACEI* angiotensin converting enzyme inhibitor, *ARB* angiotensin receptor blocker, *ASA* American Society of Anesthesiologists, *MMSE* Mini-Mental Status Examination, *NRS* numeric rating scale

^a^Smoking half a pack of cigarettes per day for at least 2 years

^b^Two drinks or more daily, or weekly consumption of the equivalent of 150 mL of alcohol

^c^Score ranges from 0 to 30, with higher score indicating better function

^d^An 11-point scale where 0 indicates no pain and 10 the worst pain

^e^An 11-point scale where 0 indicates the best possible sleep and 10 the worst possible sleep

**Table 2:** Intraoperative and postoperative management

	Placebo group (*n* = 354)	Dexmedetomidine group (*n* = 356)	*P* value
**Intraoperative data**			
Type of anesthesia			0.783
Regional	202 (57.1%)	195 (54.8%)	
General	113 (31.9%)	117 (32.9%)	
Combined regional-general	39 (11.0%)	44 (12.3%)	
Duration of anesthesia (min)	154 (105, 223)	150 (105, 215)	0.537
Intra-operative medication			
Use of midazolam	159 (44.9%)	147 (41.3%)	0.330
Midazolam (mg) ^a^	2 (1, 2)	2 (1, 2)	0.857
Use of propofol	187 (52.8%)	204 (57.3%)	0.230
Propofol (mg) ^a^	512 (205, 854)	500 (165, 784)	0.523
Use of etomidate	155 (43.8%)	147 (41.3%)	0.502
Etomidate (mg) ^a^	10 (8, 15)	10 (8, 14)	0.272
Use of dexmedetomidine	49 (13.8%)	43 (12.1%)	0.484
Dexmedetomidine (μg) ^a^	20 (20, 30)	20 (20, 30)	0.931
Use of sufentanil	164 (46.3%)	170 (47.8%)	0.704
Sufentanil (μg) ^a^	30 (18, 65)	30 (20, 53)	0.450
Use of remifentanil	88 (24.9%)	107 (30.1%)	0.121
Remifentanil (mg) ^a^	0.8 (0.5, 1.2)	0.7 (0.4, 1.1)	0.502
Use of methylprednisolone ^b^	173 (48.9%)	174 (48.9%)	0.999
Methylprednisolone (mg) ^a^	40 (40, 40)	40 (40, 40)	0.087
Use of dexamethasone ^b^	22 (6.2%)	21 (5.9%)	0.860
Use of atropine ^c^	67 (18.9%)	82 (23.0%)	0.179
Type of surgery			0.716
Joint arthroplasty	239 (67.5%)	238 (66.9%)	
Hip fracture repair	10 (2.8%)	14 (3.9%)	
Spinal surgery	105 (29.7%)	104 (29.2%)	
Duration of surgery (min)	90 (69, 145)	90 (61, 138)	0.511
Estimated blood loss (ml)	100 (50, 300)	100 (50, 250)	0.643
Total infusion (ml)	1600 (1300, 2000)	1500 (1300, 2000)	0.757
Autologous blood salvage	73 (20.6%)	68 (19.1%)	0.612
Allogeneic blood transfusion	24 (6.8%)	27 (7.6%)	0.678
ICU admission after surgery	8 (2.3%)	6 (1.7%)	0.582
**Postoperative data within 5 days**			
Consumed study drugs during PCIA ^d^			
Duration of PCIA (h)	58 ± 10	58 ± 8	0.285
Sufentanil (μg)	105 ± 35	104 ± 33	0.654
Dexmedetomidine (μg)			
Until day 1 afternoon	–-	47 ± 15	–-
Until day 2 afternoon	–-	81 ± 23	–-
Until day 3 afternoon	–-	104 ± 33	–-
Rate of dexmedetomidine (μg.kg^−1^.h^−1^)	–-	0.026 ± 0.009	–-
Supplemental analgesics within 5 days			
Use of flurbiprofen axetil	292 (82.5%)	285 (80.1%)	0.407
Flurbiprofen axetil (mg) ^a^	200 (100, 350)	250 (150, 350)	0.310
Use of parecoxib	93 (26.3%)	106 (29.8%)	0.299
Parecoxib (mg) ^a^	120 (40, 200)	120 (40, 200)	0.704
Use of loxoprofen ^e^	33 (9.3%)	37 (10.4%)	0.632
Use of oxycodone/acetaminophen ^f^	110 (31.1%)	93 (26.1%)	0.144
Use of tramadol ^g^	70 (19.8%)	61 (17.1%)	0.365
Supplemental hypnotics within 5 days			
Use of diazepam	23 (6.5%)	22 (6.2%)	0.862
Use of estazolam	18 (5.1%)	15 (4.2%)	0.581
5HT3 receptor antagonist	168 (47.5%)	169 (47.5%)	0.997

Data are number (%), median (interquartile range) or mean ± SD

*ICU* intensive care unit, *PCA* patient-controlled analgesia, *5HT3* 5-Hydroxytryptamine-3

^a^Dose in patients who received the medication

^b^For prophylaxis of postoperative nausea and vomiting and/or alleviating neuroedema

^c^Administered in combination with neostigmine, for reversal of residual neuromuscular blockade

^d^Established with 1.25 μg.ml^−1^ sufentanil and the designated study drug, either 1.25 μg.ml^−1^ dexmedetomidine or 0.9% saline, in 0.9% saline, programmed to deliver a 2-ml bolus with a lockout interval of 8 min and a background infusion of 1 ml.h^−1^

^e^Loxoprofen tablet (60 mg)

^f^Oxycodone/acetaminophen tablet (5/325 mg)

^g^Tramadol tablet (50 mg)

Postoperative delirium occurred in 26 of 354 (7.3%) of patients given placebo and in 17 of 356 (4.8%) of those given dexmedetomidine: relative risk 0.65, 95% CI 0.36 to 1.18, *P* = 0.151. In the per-protocol analysis, 26 of 347 (7.5%) of the patients given placebo and 17 of 351 (4.8%) of those given dexmedetomidine: relative risk 0.65, 95% CI 0.36 to 1.17, *P* = 0.145. Hypoactive delirium was most common in both groups (Table 3). In a post-hoc analysis, the prevalence of delirium was slightly lower in patients given dexmedetomidine [12 of 356 (3.4%)] than in those given placebos on the first postoperative day [23 of 354 (6.5%)], but the difference was not statistically significant (relative risk 0.519, 95% CI 0.262 to 1.026, *P* = 0.054; Supplemental Figure S1). Pre-defined sub-group analyses are presented in Supplemental Figure S2. No sub-group interactions were statistically significant.

**Table 3:** Effectiveness outcomes

	Placebo group (*n* = 354)	Dexmedetomidine group (*n* = 356)	RR, HR, or estimated difference (95% CI) ^a^	*P* value
**Primary outcome**				
Overall incidence of delirium	26 (7.3%)	17 (4.8%)	RR = 0.650 (0.359, 1.177)	0.151
Overall incidence of delirium (per-protocol analysis)	26 (7.5%) (*n* = 347)	17 (4.8%) (*n* = 351)	RR = 0.646 (0.356, 1.170)	0.145
**Secondary outcomes**				
Sufentanil equivalent within 5 postoperative days (μg)	107 (92, 133)	106 (89, 129)	Median D = -2.6 (-7.5, 2.4)	0.294
Use of NSAIDs within 5 postoperative days ^b^	307 (86.7%)	315 (88.5%)	RR = 1.020 (0.965, 1.078)	0.477
Length of hospital stay (day)	5 (5, 6)	5 (5, 6)	HR = 1.033 (0.891, 1.197)	0.593
Non-delirium complications within 30 days	18 (5.1%)	11 (3.1%)	RR = 0.608 (0.291, 1.268)	0.179
All-cause 30-day mortality	0 (0.0%)	0 (0.0%)	–-	> 0.999
WHOQOL-BREF, score^c^				
Physical domain	51.6 ± 14.6 [2]	55.4 ± 14.3 [5]	Mean D = 3.8 (1.6, 5.9)	0.001
Psychological domain	70.3 ± 11.1 [2]	73.1 ± 13.4 [5]	Mean D = 2.8 (1.0, 4.7)	0.002
Social relationships domain	71.9 ± 6.2 [2]	72.5 ± 5.7 [5]	Mean D = 0.6 (-0.3, 1.5)	0.176
Environment domain	61.7 ± 10.8 [2]	63.1 ± 10.4 [5]	Mean D = 1.4 (-0.1, 3.0)	0.076
TICS-m, score ^d^	28.8 ± 5.0 [5]	28.4 ± 5.0 [10]	Mean D = -0.3 (-1.1, 0.4)	0.382
**Exploratory analyses**				
Motoric subtype of delirium				0.351
None	328 (92.7%)	339 (95.2%)		
Hypoactive	20 (5.6%)	11(3.1%)		
Hyperactive	5 (1.4%)	4 (1.1%)		
Mixed	1 (0.3%)	2 (0.6%)		
Delirium requiring drug treatment ^e^	1 (0.3%)	1 (0.3%)	RR = 0.994 (0.062, 15.836)	> 0.999

Data are number (%), median (interquartile range), median (95% CI), or mean ± SD. Numbers in square brackets indicate patients with missing data

*RR* relative risk, *HR* hazard ratio, *D* difference, *NSAIDs* non-steroid anti-inflammatory drugs, *WHOQOL-BREF* the World Health Organization Quality of Life-brief version, *TICS-m* the Chinese version Telephone Interview for Cognitive Status-modified

^a^Calculated as dexmedetomidine group *vs.* or minus placebo group

^b^Include flurbiprofen axetil, parecoxib and loxoprofen

^c^Seven patients were lost to follow-up at 30 days

^d^Seven patients were lost to follow-up at 30 days; eight patients refused cognitive assessment at 30 days

^e^These two patients received dexmedetomidine

**Fig. 2:**
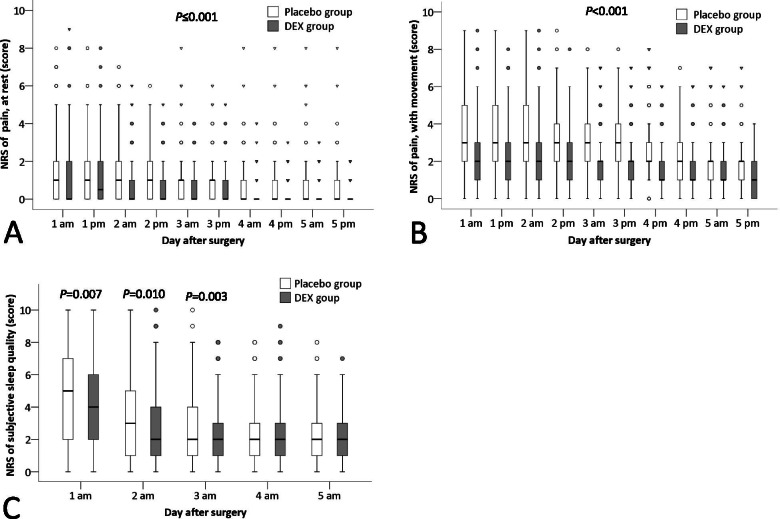
Comparison of the NRS pain score at rest (**A**) and with movement (**B**), and the NRS of subjective sleep quality (**C**) between groups. The box and whiskers plots show medians, interquartile ranges and outer ranges, and individual points mean mild outliers (○, which are outside 1.5 times of interquartile range) and extreme outliers (▽, which are outside 3 times of interquartile range). NRS, numeric rating scale. DEX, dexmedetomidine

Pain severity at rest was lower in the dexmedetomidine group than in the placebo group across the first 5 postoperative days, with the median difference being -1 to 0 points, *P* ≤ 0·001; among these, the differences were clinically significant at 3 time-points, i.e., day 1 afternoon, day 2 morning, and day 2 afternoon. Pain severity during movement was also lower in the dexmedetomidine group across the first 5 postoperative days, with a median difference -1 points, *P* < 0·001; the differences were clinically significant at all 10 time-points. Subjective sleep quality was better in patients given dexmedetomidine than placebo during the initial 3 postoperative days (day 1: median difference -1, 95% CI -1 to 0 points, *P* = 0.007; day 2: median difference 0, 95% CI -1 to 0 points, *P* = 0.010; day 3: median difference 0, 95% CI -1 to 0 points, *P* = 0.003); among these, the improvement on day 1 was clinically significant. RAAS scores were similar in dexmedetomidine and placebo patients throughout the first 5 postoperative days (Fig. 2, Supplemental Table S1).

Regarding other secondary outcomes after surgery, sufentanil equivalent dose within 5 days, use of non-steroidal anti-inflammatory drugs within 5 days, length of hospital stays, and non-delirium complications within 30 days did not differ between the two groups. No patient died within 30 days. At 30 days after surgery, physical (mean difference 3.8, 95% CI 1.6 to 5.9, *P* = 0.001) and psychological (mean difference 2.8, 95% CI 1.0 to 4.7, *P* = 0.002) components of the WHOQOL-BREF were both better in dexmedetomidine than placebo patients, but the differences were too small to be clinically important. The social and environment domains of the WHOQOL-BREF and the TICS-m score did not differ significantly (Table 3, Supplemental Table S2). The incidence of adverse events was similar in the two groups. No severe adverse events occurred during the study period (Table 4).

**Table 4:** Safety outcomes

	Placebo group (*n* = 354)	Dexmedetomidine group (*n* = 356)	*P* value
Bradycardia ^a^	1 (0.3%)	1 (0.3%)	> 0.999
Bradycardia with intervention ^b^	1 (0.3%)	1 (0.3%)	> 0.999
Hypotension ^c^	3 (0.8%)	2 (0.6%)	0.686
Hypotension with intervention ^d^	1 (0.3%)	0 (0.0%)	0.499
Tachycardia ^e^	0 (0.0%)	2 (0.6%)	0.499
Tachycardia with intervention ^b^	0 (0.0%)	1 (0.3%)	> 0.999
Hypertension ^f^	5 (1.4%)	3 (0.8%)	0.504
Hypertension with intervention ^b^	2 (0.6%)	1 (0.3%)	0.628
Hypoxemia ^g^	1 (0.3%)	1 (0.3%)	> 0.999
Hypoxemia with intervention ^h^	1 (0.3%)	1 (0.3%)	> 0.999
PCA modified due to adverse events			0.896
None	343 (96.9%)	347 (97.5%)	
Stopped temporarily ^i^	5 (1.4%)	4 (1.1%)	
Stopped permanently ^i^	6 (1.7%)	5 (1.4%)	
Postoperative nausea and vomiting	101 (28.5%)	81 (22.8%)	0.078

Data are number (%)

*PCA* patient-controlled analgesia

^a^Heart rate < 45 beats per minute

^b^Included adjustment of study drug infusion rate and/or administration of medication

^c^Systolic blood pressure < 90 mmHg, or a decreased of more than 30% from baseline

^d^Included adjustment of study drug infusion rate, intravenous fluid infusion, and/or administration of medication

^e^Heart rate > 100 beats per minute

^f^Systolic blood pressure > 180 mmHg, or an increase of more than 30% from baseline

^g^Pulse oxygen saturation < 90%

^h^Included administration of oxygen (for patients without endotracheal intubation), adjustment of ventilator setting (for patients with endotracheal intubation), and/or physical therapy

^i^Due to postoperative nausea and vomiting

## Discussion

Results of this blinded randomized trial showed that, in elderly patients following major orthopedic surgery, dexmedetomidine supplemented intravenous analgesia did not reduce delirium within 5 days; however, it improved analgesia and subjective sleep quality without increasing adverse events.

Postoperative pain, opioids, and sleep disruption each potentially contribute to delirium. Patient-controlled sufentanil combined with dexmedetomidine provided better analgesia than sufentanil alone which is consistent with previous reports [12], although opioid use was similar in each group. Dexmedetomidine also modestly improved subjective sleep quality, again consistent with previous reports [13, 30]. However, although dexmedetomidine supplemented analgesia reduced the risk of delirium by about a third, the confidence interval is wide with a potential reduction up to 64% but also with a potential increase of up to 18% and the difference is not statistically significant. The effect of dexmedetomidine supplemented analgesia in preventing delirium deserves further study in high-risk patients.

In the present study, postoperative delirium developed in 7.3% of patients in the placebo group. This incidence was lower than we expected and less than described in some reports [1–3, 5, 28], but within some recently described estimates which range from 2.2 to 10.5% [32, 33]. There are several factors potentially contributing to the relatively low delirium incidence in our patients. With the exception of the hip fracture patients (3.4% of the trial population) [33], most of our patients did not require ICU admission and were at relatively low risk for postoperative delirium, especially as many had arthroplasty [1], and 68% of the trial population had regional rather than general anesthesia [28, 30]. As expected from previous reports and our own experience [28, 30], delirium was most common on the initial postoperative day.

The largest previous trial of dexmedetomidine supplemented analgesia for prevention of delirium was by Sun and colleagues who randomized 557 non-cardiac surgical patients to analgesia with opioids alone, or opioids combined with dexmedetomidine (0.1 µg·kg^−1^·h^−1^) for the initial 48 postoperative hours [34]. Both analgesia and sleep quality improved, but the relative risk reduction for delirium was only 15% which was not statistically significant. Dexmedetomidine supplemented analgesia also failed to reduce delirium in another much smaller and seriously under-powered trial despite improved analgesia [35]. Supplemental dexmedetomidine therefore remains a reasonable sedative and analgesic, but should not be used with the expectation that it will much reduce delirium even in elderly patients recovering from major surgery.

In previous studies, postoperative administration of dexmedetomidine significantly reduced delirium in patients who remained overnight in the intensive care unit [30, 36, 37], but not those transferred to the general wards after non-cardiac surgery [34, 35]. Power was reduced in patients sent to surgical wards because they were presumably healthier and had less delirium than those who stayed in an intensive care unit (13.0 versus 22.4% in control patients) [30, 34–37]. Another reason is that for safety, ward studies used lower doses of dexmedetomidine (0.06–0.1 µg·kg^−1^·h^−1^) than those observed in monitored settings (0.1–0.7 µg·kg^−1^·h^−1^) [30, 34–37]. In the present study, we adopted a dosing regimen (mean infusion rate 0.026 µg.kg^−1^.h^−1^) even lower than in previous studies performed in general wards [34, 35], in order to avoid potential side effects including sedation [38]. To the extent that the drug’s effect is dose-dependent, less treatment effect would be expected in ward patients, again reducing trial power. However, prudence is necessary when administering dexmedetomidine in postoperative patients, especially those in the general ward. In a recent trial of 798 participants having cardiac surgery, even moderate dose dexmedetomidine (0.1–0.4 μg·kg^−1·^ h^−1^ until 24 h postoperatively) increased clinical important hypotension [39].

Orthopedic surgery is associated with substantial postoperative pain which may impair recovery by increasing complications including delirium [7]. Multimodal analgesia is thought to relieve pain after orthopedic surgery, but often insufficiently so. Most multimodal regimens do not include dexmedetomidine. In our patients, dexmedetomidine reduced NRS pain scores at rest and with movement; the changes reached or surpassed the minimal clinically important difference [40]. Our results are consistent with Shin et al. [41] who reported improved analgesia for up to 48 h. Interestingly, the analgesic benefits provided by dexmedetomidine extended beyond its biological half-life of 2 h. Specifically, analgesia was improved throughout 5 days of recovery even though the drug was always discontinued within 72 h. Supplemental dexmedetomidine thus appears to be a good strategy for relieving postoperative pain although further studies are required to confirm our findings.

Low-dose dexmedetomidine improves subjective sleep quality [13, 30]. It also improves sleep architecture by reducing stage N1 sleep, increasing stage N2 sleep, and increasing sleep efficiency [11, 38]. As might therefore be expected, we found that dexmedetomidine improved subjective sleep quality during the first 3 postoperative days. Dexmedetomidine might have improved sleep by activating endogenous sleep pathways [42]. Furthermore, good analgesia surely improves postoperative sleep [43]. In our results, dexmedetomidine did not cause excessive sedation or hemodynamic fluctuations, suggesting that the drug in current dose is a safe sedative and analgesic adjuvant.

The major limitation of this study is insufficient power. Our trial was under-powered mostly because the delirium incidence was lower than expected and because the apparent treatment effect was 35% rather than the anticipated 50%. But our results were in line with previous trials, indicating that the effect of dexmedetomidine supplemented analgesia on delirium appears to be relatively small. For safety reasons, the dose we used was relatively low; furthermore, because it was incorporated into patient-controlled analgesia, patients in pain received more of the drug. From a clinical perspective, this approach is reasonable and is often used, but differing doses does complicate interpretation of trial results. Multimodal analgesia was used in our patients, but there was no standardized pain management protocol. This also complicated the interpretation although increased the generalizability of our results. Comparison of outcomes repeated over time, such as pain severity and subjective sleep quality, increased the risk of type I errors. In our results, we did not correct for multiple outcomes. However, the differences of most of these results are robust and statistical compensation for multiplicity would not change our interpretations. Another limitation of our trial is that we did not consider long-term consequences of dexmedetomidine prophylaxis, but given the relatively small effect on delirium, it seems unlikely that any would have been observed.

In summary, supplementing sufentanil intravenous analgesia with low-dose dexmedetomidine did not significantly reduce delirium, but improved analgesia and sleep quality without provoking adverse events. The study was underpowered for the primary outcome. Dexmedetomidine remains a suitable supplement for multimodal analgesia in orthopedic surgical patients but further studies are required.

## Supplementary Information

**Additional file 1: Supplemental Figure S1.** Daily prevalence of postoperative delirium. Sample sizes differ from the first to fifth day because some patients were discharged from hospital during this period.

**Additional file 2: Supplemental Figure S2.** Forest plot assessing the effect of dexmedetomidine supplemented analgesia versus placebo in predefined subgroups. The interactions between treatment effect and predefined factors were assessed separately with logistic regression models. MMSE, Mini-Mental Status Examination.

**Additional file 3: Supplemental Table S1.** Pain, sedation, and subjective sleep quality within 5 days after surgery.

**Additional file 4: Supplemental Table S2.** Individual complications after surgery

## Data Availability

The datasets used and analyzed in the current study are available from the corresponding author upon reasonable request.

## Abbreviations

CONSORT: Consolidated Standards of Reporting Trials
RAAS: Richmond Agitation-Sedation Scale
ICU: Intensive care unit
MMSE: Mini-Mental State Examination score
NRS: Numeric Rating Scale
CAM: Confusion Assessment Method
CAM-ICU: CAM for the Intensive Care Unit
TICS-m: Telephone Interview for Cognitive Status-modified
WHOQOL-BREF: World Health Organization Quality of Life-brief version
