# Supplementary material for: Impact of dexmedetomidine supplemented analgesia on delirium in patients recovering from orthopedic surgery: A randomized controlled trial
Source: BMC Anesthesiol. 2021 Sep 13;21:223. doi: 10.1186/s12871-021-01441-3 (PMC8435562; doi:10.1186/s12871-021-01441-3)
Supplement: Supplementary file 1 — Additional file 1: Supplemental Figure S1. Daily prevalence of postoperative delirium. Sample sizes differ from the first to fifth day because some patients were discharged from hospital during this period. [file 12871_2021_1441_MOESM1_ESM.docx]

| Number at risk |  |  |  |  |  |
| --- | --- | --- | --- | --- | --- |
| Placebo group | 354 | 352 | 325 | 271 | 203 |
| Dexmedetomidine group | 356 | 354 | 321 | 264 | 203 |

**Supplemental Figure S1.** Daily prevalence of postoperative delirium. Sample sizes differ from the first to fifth day because some patients were discharged from hospital during this period.
