# Supplementary material for: Impact of dexmedetomidine supplemented analgesia on delirium in patients recovering from orthopedic surgery: A randomized controlled trial
Source: BMC Anesthesiol. 2021 Sep 13;21:223. doi: 10.1186/s12871-021-01441-3 (PMC8435562; doi:10.1186/s12871-021-01441-3)
Supplement: Supplementary file 2 — Additional file 2: Supplemental Figure S2. Forest plot assessing the effect of dexmedetomidine supplemented analgesia versus placebo in predefined subgroups. The interactions between treatment effect and predefined factors were assessed separately with logistic regression models. MMSE, Mini-Mental Status Examination. [file 12871_2021_1441_MOESM2_ESM.pdf]

	Dexmedetomidine group	Placebo group	Relative risk for postoperative delium (95% CI)	Interaction *P* value
	Events/total (*n*/*N*)			
**Study centre**				0.827
Centre 1	6/212	8/210	0.743 (0.262 to 2.104)	
Centre 2	11/144	18/144	0.611 (0.299 to 1.248)	
**Age (year)**				0.269
<70	9/165	9/171	1.036 (0.422 to 2.546)	
≥70	8/191	17/183	0.451 (0.199 to 1.019)	
**Sex**				0.700
Male	5/116	6/113	0.603 (0.301 to 1.205)	
Female	12/240	20/241	0.812 (0.255 to 2.585)	
**Preoperative MMSE (score)**				0.528
≥26	5/208	6/217	0.869 (0.269 to 2.805)	
<26	12/148	20/137	0.555 (0.282 to1.093)	
**Surgery type**				0.710
Joint surgery	12/252	20/249	0.593 (0.296 to 1.187)	
Spinal surgery	5/104	6/105	0.841 (0.265 to 2.672)	
**Anaesthesia type**				0.904
Regional only	12/195	19/202	0.654 (0.326 to 1.311)	
General/combined	5/161	7/152	0.674 (0.219 to 2.079)	

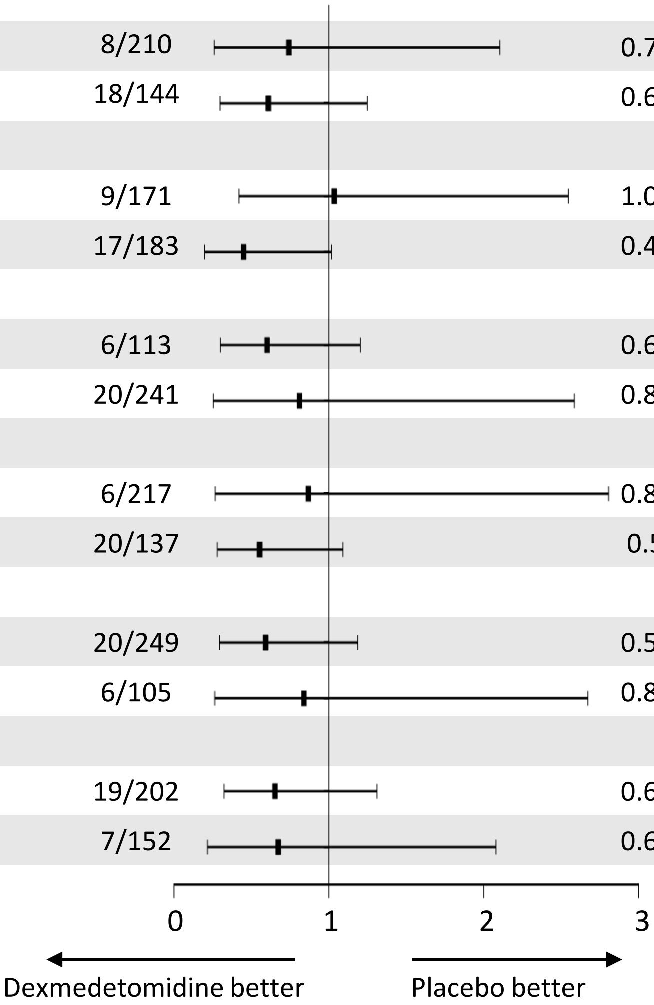

Supplemental Figure S2. Forest plot assessing the effect of dexmedetomidine supplemented analgesia versus placebo in predefined subgroups. The interactions between treatment effect and predefined factors were assessed separately with logistic regression models. MMSE, Mini-Mental Status Examination.
