# Supplementary material for: Impact of dexmedetomidine supplemented analgesia on delirium in patients recovering from orthopedic surgery: A randomized controlled trial
Source: BMC Anesthesiol. 2021 Sep 13;21:223. doi: 10.1186/s12871-021-01441-3 (PMC8435562; doi:10.1186/s12871-021-01441-3)
Supplement: Supplementary file 3 — Additional file 3: Supplemental Table S1. Pain, sedation, and subjective sleep quality within 5 days after surgery. [file 12871_2021_1441_MOESM3_ESM.docx]

**Supplemental Table S1.** Pain, sedation, and subjective sleep quality within 5 days after surgery.

|  | Placebo group (*n*=354) | Dexmedetomidine group (*n*=356) | Median difference (95% CI) ^a^ | *P* value |
| --- | --- | --- | --- | --- |
| NRS of pain after surgery, at rest (score) |  |  |  |  |
| Day 1 morning | 1 (0 to 2) | 0 (0 to 2) | 0 (-1 to 0) | **<0.001** |
| Day 1 afternoon | 1 (0 to 2) | 0.5 (0 to 2) | -1 (-1 to 0) | **<0.001** |
| Day 2 morning | 1 (0 to 2) | 0 (0 to 1) | -1 (-1 to 0) | **<0.001** |
| Day 2 afternoon | 1 (0 to 2) | 0 (0 to 1) | -1 (-1 to 0) | **<0.001** |
| Day 3 morning | 1 (0 to 1) | 0 (0 to 1) | 0 (-1 to 0) | **<0.001** |
| Day 3 afternoon | 1 (0 to 1) | 0 (0 to 1) | 0 (0 to 0) | **<0.001** |
| Day 4 morning | 0 (0 to 1) | 0 (0 to 1) | 0 (0 to 0) | **<0.001** |
| Day 4 afternoon | 0 (0 to 1) | 0 (0 to 0) | 0 (0 to 0) | **<0.001** |
| Day 5 morning | 0 (0 to 1) | 0 (0 to 0) | 0 (0 to 0) | **<0.001** |
| Day 5 afternoon | 0 (0 to 1) | 0 (0 to 0) | 0 (0 to 0) | **0.001** |
| NRS of pain after surgery, with movement (score) |  |  |  |  |
| Day 1 morning | 3 (2 to 5) | 2 (1 to 3) | -1 (-1 to -1) | **<0.001** |
| Day 1 afternoon | 3 (2 to 5) | 2 (1 to 3) | -1 (-1 to -1) | **<0.001** |
| Day 2 morning | 3 (2 to 5) | 2 (1 to 3) | -1 (-2 to -1) | **<0.001** |
| Day 2 afternoon | 3 (2 to 4) | 2 (1 to 3) | -1 (-1 to -1) | **<0.001** |
| Day 3 morning | 3 (2 to 4) | 2 (1 to 2) | -1 (-1 to -1) | **<0.001** |
| Day 3 afternoon | 3 (2 to 4) | 2 (1 to 2) | -1 (-1 to -1) | **<0.001** |
| Day 4 morning | 2 (2 to 3) | 1 (1 to 2) | -1 (-1 to -1) | **<0.001** |
| Day 4 afternoon | 2 (1 to 3) | 1 (1 to 2) | -1 (-1 to -1) | **<0.001** |
| Day 5 morning | 2 (1 to 2) | 1 (1 to 2) | -1 (-1 to 0) | **<0.001** |
| Day 5 afternoon | 2 (1 to 2) | 1 (0 to 2) | -1 (-1 to 0) | **<0.001** |
| RASS of sedation after surgery (score) |  |  |  |  |
| Day 1 morning | 0 (0 to 0) | 0 (0 to 0) | 0 (0 to 0) | 0.857 |
| Day 1 afternoon | 0 (0 to 0) | 0 (0 to 0) | 0 (0 to 0) | 0.705 |
| Day 2 morning | 0 (0 to 0) | 0 (0 to 0) | 0 (0 to 0) | 0.778 |
| Day 2 afternoon | 0 (0 to 0) | 0 (0 to 0) | 0 (0 to 0) | 0.591 |
| Day 3 morning | 0 (0 to 0) | 0 (0 to 0) | 0 (0 to 0) | 0.709 |
| Day 3 afternoon | 0 (0 to 0) | 0 (0 to 0) | 0 (0 to 0) | 0.808 |
| Day 4 morning | 0 (0 to 0) | 0 (0 to 0) | 0 (0 to 0) | 0.573 |
| Day 4 afternoon | 0 (0 to 0) | 0 (0 to 0) | 0 (0 to 0) | 0.996 |
| Day 5 morning | 0 (0 to 0) | 0 (0 to 0) | 0 (0 to 0) | 0.417 |
| Day 5 afternoon | 0 (0 to 0) | 0 (0 to 0) | 0 (0 to 0) | 0.082 |
| NRS of subjective sleep quality after surgery (score) |  |  |  |  |
| Day 1 morning | 5 (2 to 7) | 4 (2 to 6) | -1 (-1 to 0) | **0.007** |
| Day 2 morning | 3 (1 to 5) | 2 (1 to 4) | 0 (-1 to 0) | **0.010** |
| Day 3 morning | 2 (1 to 4) | 2 (1 to 3) | 0 (-1 to 0) | **0.003** |
| Day 4 morning | 2 (1 to 3) | 2 (1 to 3) | 0 (0 to 0) | 0.292 |
| Day 5 morning | 2 (1 to 3) | 2 (1 to 3) | 0 (-1 to 0) | 0.054 |

Data are median (interquartile range). *P* values in bold indicate <0.05.

^a^ Calculated as dexmedetomidine group *vs.* or minus placebo group.
