# Supplementary material for: Impact of dexmedetomidine supplemented analgesia on delirium in patients recovering from orthopedic surgery: A randomized controlled trial
Source: BMC Anesthesiol. 2021 Sep 13;21:223. doi: 10.1186/s12871-021-01441-3 (PMC8435562; doi:10.1186/s12871-021-01441-3)
Supplement: Supplementary file 4 — Additional file 4: Supplemental Table S2. Individual complications after surgery [file 12871_2021_1441_MOESM4_ESM.docx]

**Supplemental Table S2.** Individual complications after surgery

|  | Placebo group (*n*=354) | Dexmedetomidine group (*n*=356) | *P* value |
| --- | --- | --- | --- |
| Stroke ^a^ | 3 (0.8%) | 1 (0.3%) | 0.373 |
| Circulatory insufficiency ^b^ | 1 (0.3%) | 0 (0.0%) | 0.499 |
| Acute myocardial infarction ^c^ | 2 (0.6%) | 0 (0.0%) | 0.249 |
| New onset arrhythmia ^d^ | 0 (0.0%) | 1 (0.3%) | >0.999 |
| Pulmonary infection ^e^ | 1 (0.3%) | 0 (0.0%) | 0.499 |
| Acute renal injury ^f^ | 1 (0.3%) | 0 (0.0%) | 0.499 |
| Wound complication ^g^ | 4 (1.1%) | 4 (1.1%) | >0.999 |
| Urinary tract infection ^h^ | 1 (0.3%) | 0 (0.0%) | 0.499 |
| Pulmonary embolism ^i^ | 1 (0.3%) | 2 (0.6%) | >0.999 |
| Deep vein thrombosis ^j^ | 4 (1.1%) | 3 (0.8%) | 0.725 |

Data are number (%).

^a^ Persisted new focal neurologic deficit and confirmed by neurologic imaging.

^b^ Requirement of inotropic agents or vasoconstrictors for more than 24 hours after surgery.

^c^ Concentration of cardiac troponin I exceed the diagnostic criteria for myocardial infarction as well as new Q waves (lasts for 0.03s) or continuous (4 days) abnormal ST-T segment.

^d^ Atrial fibrillation and paroxysmal supraventricular tachycardia in this study and necessitating medical treatment.

^e^ New infiltrate on chest radiograph combined with temperature over 38°C and leucocytosis.

^f^ Confirmed by nephrologist according to the KIDGO guideline for acute kidney injury.

^g^ Delayed healing, persistent exudation, flap necrosis, debridement and secondary suture required.

^h^ Confirmed by urinalysis and urine culture and necessitated antibiotic therapy.

^i^ Confirmed by computed tomography pulmonary angiography or pulmonary ventilation/perfusion scan and necessitated anticoagulation therapy.

^j^ Confirmed by deep vein ultrasonography and necessitated anticoagulation therapy.
